# Factors associated with adverse pregnancy outcomes and perceptions of risk factors among reproductive age women in Soba LGA, Kaduna State 2013

**DOI:** 10.11604/pamj.2016.25.111.8739

**Published:** 2016-10-25

**Authors:** Aishatu Abubakar Sadiq, Gabriele Poggensee, Patrick Nguku, Kabir Sabitu, Aisha Abubakar, Thandi Puone

**Affiliations:** 1 Nigeria Field Epidemiology and Laboratory Training Programme, Abuja, Nigeria; 2 Department of Community Medicine, Ahmadu Bello University Zaria, Kaduna, Nigeria; 3 University of western cape, South Africa

**Keywords:** Antenatal, pregnancy, eclampsia, Nigeria

## Abstract

**Introduction:**

Maternal mortality defined as deaths due to complications of pregnancy or childbirth remains a public health concern. Although statistics show a decline in maternal mortality ratio from 380 deaths to 210 deaths per 100,000 live births from1990 to 2013, in Sub-Saharan Africa, maternal mortality rates remain unacceptably high. Maternal mortality In Nigeria is currently 560/100,000 live births. This study was conducted to identify the associated risk factors and perceptions of adverse pregnancy outcomes among reproductive age women in Soba local government area (LGA).

**Methods:**

A 1:1 unmatched case control study with 138 respondents was used. Cases were women aged 15-49 years with a history of adverse pregnancy outcome. Controls: 15-49 years without a history of adverse outcomes. Adverse outcomes were: pregnancy induced hypertension and spontaneous abortions. Anthropometric measurements and blood pressure were taken. Six focus group discussions (FGDs) with grandmothers, mothers and teenagers were used to explore perceptions. Quantitative data was analyzed using Epi-info version 3.5.3. Qualitative data analyzed by thematic approach.

**Results:**

The median age of cases was: 25 years (Range: 16-44years), Median age of controls: 27 years (Range: 16-43years). Commencement of Antenatal care (ANC) attendance <4months (adjusted odds ratio (AOR): 0.32; 95% CI: 0.12-0.81) and Number of pregnancies ≥4 (AOR: 5.02; 95% CI: 1.97-12.82) were found to be associated with adverse outcomes.

**Conclusion:**

Risk factors associated with outcomes are multiple pregnancies and delayed commencement of antenatal care. There was poor perception of adverse pregnancy outcomes. We recommended frequent community health talks, early commencement of antenatal and Utilization of Family planning services.

## Introduction

There is a vast difference in pregnancy outcomes between high income and many middle and low income countries. In many low-income countries, the maternal mortality ratio is 100-fold greater than in high-income countries (HIC). Even if both mother and infant survive, pregnancy complications or problems at delivery or during the neonatal period can lead to severe maternal or infant morbidity [1]. According to the world health organization (WHO) Nigeria had the highest estimated number of maternal deaths in Africa, and ranked eighth in the sub-Saharan region behind Angola, Chad, Liberia, Niger, Rwanda, Sierra Leone and Liberia [2]. 75% percent (27,750) of these maternal deaths are attributable to direct obstetric complications, such as infection, toxemia, and unsafe induced abortion. Studies have shown these complications can be prevented; they cause deaths only because of severe socioeconomic deprivations that are present in these countries [3]. Hypertensive disorders are among the most common causes of maternal and perinatal mortality .High blood pressure (BP) complicates approximately 10% of all pregnancies. In Northern Nigeria eclampsia has been reported as the leading cause of maternal mortality [4]. This is attributed to cultural practices leading to early child bearing in the wake of poor maternity care services that are grossly underutilized. In a recent report from Maiduguri North eastern Nigeria, eclampsia accounted for 46.4% of all maternal death [4, 5].

The disparity in pregnancy outcomes between high and low income countries has been attributed to poor antenatal care received by women in low income countries [6]. Women who receive prenatal care are appropriately screened for conditions, such as hypertension, diabetes, anemia, Rhesus disease, and syphilis; and are appropriately treated; have reduced rates of stillbirth and of neonatal and maternal mortality. Barriers to receiving prenatal care for lower-SES women may include inability to pay for otherwise available services, as well as failure to seek services because of prior negative experiences, lack of transportation and depression [7]. Preconception care (PCC) is associated with improved pregnancy outcomes. The traditional ante-natal care model used in Sub-Saharan Africa including Nigeria neglects the most critical time of embryonic development, which often occurs before a woman knows she is pregnant [8]. Preconception health is a woman´s health before she becomes pregnant. It is a set of interventions that aim to identify and modify biomedical, behavioral and social risks to a woman’s health or pregnancy outcome through prevention and management. It includes giving protection, managing conditions and avoiding exposures known to be teratogenic [9]. The evidence increasingly points to preconception care before pregnancy to improve women´s health, and better pregnancy outcomes for the mother and newborn [10, 11]. Adverse pregnancy outcomes are influenced by a myriad of biological, social and environmental factors. Numerous studies have found that socioeconomic status and income inequality are correlated with birth outcomes. A variety of other social factors have been linked to poor birth outcomes, including maternal education, marital status, pregnancy intention and teenage pregnancy [12]. Other factors such as maternal obesity, maternal co morbidities and genetic vulnerabilities have also each been linked to poor pregnancy outcomes [13, 14]. We conducted a study to assess risk factors that are associated with adverse pregnancy outcomes among reproductive age women in Soba Local Government area (LGA). We also explored women’s perceptions about these risk factors.

## Methods

**Setting:** The study was undertaken in Soba local government area (LGA) one of 23 LGAs within the state. It is located in the northern part of Kaduna state. It has a total population of 238,719 comprising predominantly traders, cattle rearers and farmers. Islam is the main religion practiced by the inhabitants. There are no tertiary health facilities or specialist hospitals in this LGA, however there are 2 primary health care (PHC) centers, 1 private clinic and multiple patent medical stores.

**Study design:** This was a 1:1 unmatched case-control study comparing identified risk factors for adverse pregnancy outcomes between selected cases and control groups. In addition a qualitative approach was used to gather information on the perceptions about significant health problems, preventive measures and risk factors. Using six focus group discussions (FGDs) conducted in Hausa Language, audio and written records were obtained. Respondents were grandmothers, mothers and teenage females each in two homogenous groups. Each focus group discussion lasted 45minutes-1 hour facilitated by a moderator. Data were transcribed and translated into English and analyzed using a thematic approach.

**Study population:** The study population comprised of all reproductive women aged (15-49years) with previous history of one or more pregnancies and residing in the study area during the survey. Eligible women found too sick to be interviewed or not permanent residents in the study area were excluded.

**Sample size calculation and sampling:** The fleiss formula for unmatched case control studies in Epi info statistical software 3.5.3 was used to calculate the sample size. Based on the following assumptions: Two Sided confidence interval (1-a) of 95% (95% CI), power of 80% was chosen to detect an odds ratio (OR) of 3. The calculated sample size was 138 (69 cases: 69 controls). The ward was selected randomly from the total 10 wards in the LGA. Systematic sampling was used to select study units. Women who met the criteria were purposefully selected till the required sample size was achieved. Independent variables were age, obesity (BMI >30), education, income, occupation and height. miscarriages (spontaneous abortion) and pregnancy induced hypertension/eclampsia were dependent/outcome variables used in this study. Cases included reproductive aged women (15-49years) with a history of adverse pregnancy outcome in the past 5 years and permanently residing in the study area. Controls were reproductive aged women (15-49years) with no history of adverse pregnancy outcome in the past 5 years and permanently residing in the study area.

**Data collection:** A pre-tested structured interviewer administered questionnaire used to collect respondent’s biodata; Section A of the questionnaire obtained information on respondents’ biodata; pre-selected risk factors and duration of exposure; pregnancy outcomes of interest and respondents’ current health status. Variables of interest included age, obesity (BMI >30), education, income, occupation and height as independent variables. Miscarriages (Spontaneous abortion) and pregnancy induced hypertension/eclampsia were dependent/outcome variables. For the qualitative component of the study, six focus group discussions (FGDs) were conducted in Hausa Language to explore the perceptions about significant health problems, preventive measures and risk factors. A Focus group discussion guide was used to facilitate discussions with homogenous groups of mothers, teenagers and grandmothers (2 groups each) purposely selected using convenience sampling. Discussions were audio recorded and written notes were taken. Data were transcribed and translated into English. Height was measured in meters (m) using standiometer and weight in kilograms (Kg) while wearing light clothing using calibrated weighing scales. Blood pressure measurements (mmHg) were taken using manual sphygmomanometers. Body mass indices were subsequently calculated.

**Statistical analysis:** Data was analyzed using Epi info version 3.6.3 statistical software (CDC Atlanta, Georgia, U.S.A). Univariate, bivariate and multivariate analysis was performed. Odds ratios and 95% confidence intervals were used to assess association between risk factors and pregnancy outcome. A p-value of ≤ 0.05 was considered significant. Qualitative data was analyzed using thematic approach.

**Ethical approval:** Ethical clearance was obtained from the ethical committee within the Kaduna state ministry of health before commencement of the study. The provisions of the HELSINKI declaration were respected: Respect for persons and their human rights: informed consent was obtained from all participants and confidentiality was maintained, Beneficence: The study had a scientific design (case-control) which answered the study question and the risks to the participants were proportional to the benefits, justice: the benefits and the harms from the research were distributed fairly in the population and all potential participants had equal chance of benefiting from the research and non-maleficense: no therapies were with-held that are of known benefit and are generally available.

## Results

**Figure 1:**
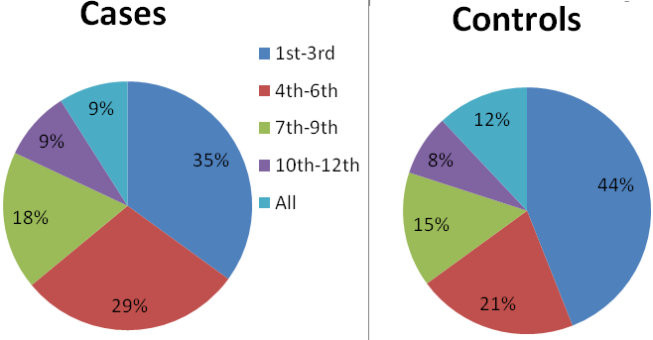
Pregnancies booked at health facilities among respondents

**Socio-demographic characteristics of respondent’s are:** Table 1 shows relative homogeneity of variables among cases and controls. A majority 46.4% of cases and 47% of controls were in the age-group 25-34 years. The median age of cases is 25 years; range (16-44 years), median age of controls 27 years; range (16-43 years). Forty two percent of cases and 52.2% of controls were unemployed. Informal education was reported 49.3% of cases and 42% of controls, with only 1.4% of cases and 11.6% of controls having attained tertiary education. 98.6% of both cases and controls were Muslims. Only 2.9% of controls were non-Hausas i.e. Igbira and Igala. Majority of both cases and controls 98.6% were married. Table 2 shows the association of risk factors to adverse pregnancy outcomes. Early ante-natal care attendance < 4 months: OR; 0.4 (CI: 0.2-0.99) and height < 1.52 meters: OR; 0.2 (CI: 0.1-0.7) were found to be protective. Pregnancies ≥4: OR; 5.6 (CI: 2.6-12.6) was found to significantly increase the risk of developing the outcomes; pregnancy induced hypertension/miscarriages. Other risk factors such as education, Income, obesity and age showed no significant association with pregnancy induced hypertension/miscarriages. Multivariate unconditional logistic regression analysis (Table 3) while controlling for age including identified risk factors with p-values of <0.25 from bivariate analysis revealed: early ante-natal care attendance < 4 months: AOR; 0.32 (CI: 0.12-0.81) was significantly protective while number of pregnancies ≥4: AOR; 5.02 (CI: 1.97-12.82) was found to increase the risk of developing the adverse outcomes (Figure 1).

**Table 1:** Socio-demographic characteristics of respondents (n=138)

Variable	Case: No (%)	Control: No (%)
**Age-group (Years):**		
15-24	13 (18.8)	28 (40.6)
25-34	32 (46.4)	33 (47.8)
35-44	21 (30.4)	7 (10.1)
>44	3 (4.4)	1 (1.5)
**Occupation:**		
Civil servant	3 (4.4)	7 (10.1)
Farmer	2 (2.9)	1 (0.0)
Trader	18 (26.1)	7 (10.1)
Craftswoman	17 (24.6)	19 (27.6)
Unemployed	29 (42.0)	36 (52.2)
**Educational level**		
Primary	23 (33.3)	24 (34.8)
Secondary	17 (15.9)	8 (11.6)
Tertiary	1 (1.4)	8 (11.6)
Informal	34 (49.3)	29 (42.0)
**Marital status**		
Married	68 (98.6)	68 (98.6)
Divorced	0 (0.0)	1 (1.4)
Widowed	1 (1.4)	0 (0.0)
**Religion**		
Islam	68 (98.6)	68 (98.6)
Christianity	1 (1.4)	1 (1.4)
**Tribe**		
Hausa	69 (100)	67 (97.1)
Other	0 (0.0)	2 (2.9)

Other Tribes: **Igbira and Igala**

**Table 2:** Risk factors for adverse pregnancy outcomes among respondents (n=138)

Variable	Cases N (Col %)	Controls N (Col %)	OR (95% CI)	P-Value
**Age first pregnancy**			1.5 (0.6-4.0)	0.37
<18	59 (85.5)	55(79.7)		
≥18	10(14.5)	14(20.3)		
**Age last pregnancy**			0.5 (0.1-1.9)	0.17
<35	59 (85.5)	64(92.7)		
≥35	10(14.5)	5(7.3)		
**BMI**			0.9 (0.3-2.4)	0.82
≥30	11(15.9)	12(17.4)		
<30	58(84.1)	57(82.6)		
**Height**			**0.2 (0.1-0.7)**	**0.004**
<1.52	**4 (5.8)**	**16(23.2)**		
≥1.52	**65(94.2)**	**53(76.8)**		
**ANC attendance**			**0.4 (0.2-0.99)**	**0.03**
<4mths	**58 (84.1)**	**47(68.1)**		
≥4 months	**11(15.9)**	**22(31.9)**		
**Number of pregnancies**			**5.6 (2.6-12.6)**	**0.00**
≥4	**50 (74.5)**	**22(31.9)**		
<4	**19(25.5)**	**47(68.1)**		
**Education**			1.2 (0.6-2.5)	0.61
Tertiary/secondary/Primary	37 (53.6)	40(58.0)		
Informal	32(46.4)	29(42.0)		
**Occupation**			1.4 (0.7-2.9)	0.31
Employed	40 (58.0)	34(49.3)		
Unemployed	29(42.0)	35(50.7)		
**Monthly income**			2.2 (0.7-7.1)	0.13
<5,000	63 (91.3)	57(82.6)		
≥5,000	6(8.7)	12(17.4)		
**Source of medical care**			0.7 (0.3-1.4)	0.20
Hospital/Clinic	38 (55.1)	45(65.2)		
Other	31(44.9)	24(34.8)		
**Pre-conception care (PCC)**			Undefined	Undefined
Yes	0(0.0)	0(0.0)		
No	69(100.0)	69(100.0)		

**Table 3:** Risk factors (Logistic Regression) for adverse pregnancy outcomes among respondents (n=138)

Variables	Adjusted Odds Ratio(aOR)	95% Confidence interval	P-value
**Height grouped**		0.40-1.80	0.38
≥1.52	0.86		
<1.52	1 (reference)		
**ANC attendance**		0.12-0.81	**0.02**
Yes(<4months)	0.32		
No(≥4months)	1(reference)		
**Number of pregnancies**		1.97-12.82	**0.00**
≥4	5.02		
<4	1(reference)		
**Age last pregnancy**		0.13-2.77	0.52
<35 years	0.60		
≥35	1(reference)		

### Results from focus group discussions

The main themes that emerged from focus group discussions were:

Health problems: commonly experienced problems during pregnancy were hypertension with seizures and miscarriages. Ulcers, leg pain and asthma were also mentioned by some respondents. Participants mentioned poor health facility staffing and financial constraints as obstacles to attaining necessary health care for early symptoms. However others believed pregnancy complications are more common with primigravidae. Utilization of family planning and female economic empowerment via job creation and skills acquisition were believed to reduce healthcare problems.

*‘’For the women our worst health problem is fits during pregnancy. Other common ones are raised blood pressure and malaria.’’* Mother-Maigana ward.

**Reasons for none or poor attendance to health facility:** Participants did not regularly attend antenatal clinics for all pregnancies, as antenatal care was thought to be necessary for either early or late pregnancies that are associated with complications. Home delivery was largely practiced as a personal preference. However in a few cases due to spousal refusal to pay for health care delivery. Majority of respondent stated that women often book a pregnancy only when it starts to show and only go to the health facility thereafter in the case of ill health. A large number of women admitted to booking pregnancies at the health facility but opting for home delivery for privacy.

*‘’They should go to the hospital only when they have problems with pregnancy. It costs too much to visit regularly.’’* Grandmother-Maigana ward .

The influence of in-laws in the choice of pregnancy and delivery site was identified by the majority of participants as influencing their decision making. An absence of pre-conception care was stated by all respondents as a problem as most pregnancies were generally believed to be unplanned.

**Risk perceptions:** The most common belief was that recurrent pregnancies (multiparity) especially women having more than 5 pregnancies increased the risk of bleeding, hypertension, seizures and miscarriages.

*‘’Having too many children weakens a woman and leads to problems with pregnancies and child-birth.’’* Grand-mother-Maigana ward.

Seizures were believed to occur more frequently among young girls and women whose mothers had problematic pregnancies.

*‘’Young girls who marry before they are 15 years always end up with fits and hypertension in pregnancy.’’*Teenager-Maigana Ward.

Extremely young mothers <13 years and women older than 40 years having babies were considered to experience more adverse outcomes than others. Some participants stated that the use of family planning especially by older women was believed to reduce health problems in pregnancy as it give their bodies time to rest following the stress of child birth. Poor nutrition and lack of adequate care were also mentioned as causes of complicated pregnancies. Women who stayed healthy and kept fit through house hold chores and occasional farming were said to have better pregnancy and delivery outcomes than those with sedentary lifestyles.

*‘’If you stay lazy and idle while pregnant you will have health problems. A woman should move around and clean her surroundings so her baby can fit well for delivery.’’* Mother-Maigana ward.

**Preventive measures:** Delaying early marriage, provision of free maternal health services and frequent antenatal care were considered key preventive measures. Responses on availability and utilization of pre-conception care and counseling were all negative as no participant had ever received pre-conception care and counseling from health personnel. However willingness to use these services if available was all positive with majority stating they would welcome the introduction of pre-conception care into their communities. A large number of participants also stated a need to influence husbands into permitting antenatal visits and providing funds for treatment. No participant had ever received pre-conception care and counseling from healthcare personnel. A willingness to utilize such care was expressed by the respondents.

*‘’If I can be helped to have a safe pregnancy and easy delivery, I will accept pre-conception care and counseling before I start having babies.”* Mother-Maigana ward.

## Discussion

This study assessed the risk factors associated with adverse pregnancy outcomes among women of reproductive age in Soba Local Government area. It also explored women’s perceptions about these risk factors. Factors that were associated with adverse pregnancy outcomes were multiparous women were twice more likely to develop hypertension in pregnancy and experience spontaneous abortions. Women who attended ANC had better pregnancy outcomes. In the current study we found adverse pregnancy outcomes were associated with pregnancy at an early age, multiparity and prolonged reproductive period. Women in focus group discussions also believed adverse pregnancy outcome was related to pregnancy at an early age. Previous facility based studies in the country reported that grand-multiparity was associated with high perinatal and maternal morbidity and mortality among women aged 30-35 years [15]. Contrary to these findings our study found adverse pregnancy outcomes among younger multiparous women 20-29 years. Women who participated in our study had a mean age of 28.6 years for cases and 23.5 years for controls, suggesting early and continuous childbearing peaking in the 30s. There is therefore a need to plan and implement preventive measures early prior to commencement of reproduction in the early teenage years. Focus group discussions also revealed that multiparous women were believed to have a higher chance of having pregnancy and delivery complications than those with lower parities. Focus group discussions also revealed lack of availability and access to pre-conception care was a contributory factor in adverse pregnancy outcomes. These findings are in line with those reported in a facility based study of 236 women who had not received preconception care or counseling showed that 36.4% of pregnancies had unfavorable outcomes [16]. The absence of preconception care and counseling result in failure to identify relevant risk factors and institute timely interventions such as treatment of co-morbities and dietary modifications. While the new model of ante-natal care recommended by the World Health Organization (WHO) requires 4 antenatal visits, which should be started in the first trimester. Findings from FGDs revealed that most women start antenatal care late and do not receive proper care in all pregnancies. If women book late, they miss the crucial recommended first visit of <16 weeks which is used to screen and implement preventive and future management plans to prevent adverse pregnancy outcomes.

Several socio-demographic characteristics in this study population have been reported previously to favor multiparity; pregnancy among females with no formal education, poverty and decreased educational engagement. These confer a two-fold risk of multiparity, preterm delivery and early pregnancy [17]. Health conditions identified among respondents were acute and not associated with pregnancy outcomes. These included peptic ulcer disease, skin allergies, recurrent headaches and cough. Obesity though implicated in the etiology of adverse pregnancy outcomes showed no significant association in this study. This may be attributed to the age distribution of the respondents and the rural nature of the study area. Studies have shown adults aged 60 years and above are more likely to be obese [18]. Regional studies within the country have also shown a significant association between increasing age and obesity. Though grand-multiparity i.e. having greater than 4 deliveries was also implicated, the age of multiparous women was between 50-59 years. Our study showed multiparity among younger women 20-29 years with low body mass indices and greater than four deliveries. The national prevalence of obesity in Nigeria is 8% although changing lifestyles, increased urbanization, high calorie consumption and reduced physical activity have produced higher values in urban studies. A Nigerian study in a semi-urban community showed obesity increasing across age gradient from young to old adults; peaking in the 60-69 year age group [19]. The strength of our study included low rates of non-response (1.5%). Furthermore the use of a mixed method of data collection both quantitative and qualitative increases the validity of our results. Nonetheless, this study had several limitations. Cases were more likely than controls to remember history of pregnancies (Recall bias). Confirmatory information on pregnancies was obtained from care-givers during pregnancy and female relatives where available (Sister-hood method). Study participants may have been unable to provide accurate details of unbooked pregnancies. We attempted to obtain further information from spouses who were present and co-habitants in large compound houses. We encountered a lack of detailed and accurate Antenatal attendance cards for verification of pregnancy/ delivery information. Supplementary information was however obtained from co-habitants and Traditional midwives. Older women residing in same households and streets who traditionally serve as initial care-givers were used to collaborate ante-natal and delivery information. Due to the conservative nature of rural communities especially in the Northern part of the country our findings have public-health policy and programmatic implications, especially in promoting maternal health and child health. We recommended the following.

**The state government:** Introduce reproductive and conception health training in the district primary schools. To enable young Girls who marry before secondary education have basic knowledge on how to stay healthy while pregnant. Provide adequate drugs and upgrade maternal health and family planning services at the hospitals and clinics in the local government area. Introduce training of healthcare personnel and their trainers in pre-conception care and counseling to enable effective ‘’step-down’’ of training and service provision to rural communities and Increase deployment of trained healthcare personnel to the local government health facilities.

**The local government:** Establish periodic house to house visits by health care personnel to interact with women on health issues especially conception and pregnancy (outreach), Establish and sustain regular community based health talks on maternal health. Efforts should be made to arrange a suitable and secluded venue to enable the conservative female majority interact fully during sessions. Increase deployment of supplementary trained healthcare personnel such as community health extension workers to the local government health facilities and Design and distribute information materials in the local language on pre-conception care, pregnancy care and relevant delivery information.

## Conclusion

Early commencement of antenatal care i.e. before four months of gestation and multiparity are associated with adverse pregnancy outcomes. Women in this rural community show early commencement of reproduction and prolonged reproductive periods. The rural setting and investigation of pre-conceptional/ pregnancy history will generate information at the community level contributing to evidence which shows most determinants of birth outcomes exist prior to conception. Results of this study will provide information for use in planning intervention strategies to improve maternal health. Establishment of preconception screening and counseling will assist in reducing the occurrence of these undesirable outcomes.

### What is known about this topic

- Most risk factors for adverse pregnancy outcomes exist before pregnancy occurs;
- Multiparity contributes to risk of pregnancy induced hypertension.

### What this study adds

- Information on specific risk factors associated with adverse outcomes in rural communities;
- Community perceptions and understanding of preconception, antenatal and delivery care.

## References

[1] Kassebaum NJ, Bertozzi-villa A, coggeshall MS (2014). Global, regional, and national levels and causes of maternal mortality during 1990-2013: A systematic analysis of the global burden of disease study. Lancet..

[2] Kramer MS (2003). The epidemiology of adverse pregnancy outcomes: McGill University press Faculty of medicine, Montreal. Journal of nutrition..

[3] Geronimus AT (1996). Black/white differences in the relationship of maternal age to birth weight: a population-based test of the weathering hypothesis. Soc Science Medical Journal..

[4] Kullima AA, Kawuwa MB, Audu BM (2009). Trends in maternal mortality in a tertiary institution in Northern Nigeria. Annual African Medical..

[5] Mairiga AG, Salihi W (2009). Maternal mortality at specialist Hospital Bauchi, Northern Nigeria. East African Journal Medicine..

[6] Gardosi J, Maternal and Child Health Research Consortium, Ed: 8th annual report (2001). Clinical implications of ‘unexplained’ stillbirths. Confidential Enquiry into stillbirths and Deaths in infancy..

[7] Gardosi J, Kaddy SM, McGeown P (2005). Classification of stillbirth by relevant condition at death (ReCoDe): Population based cohort study. British Medical Journal..

[8] Atrash H (2006). Preconception care for improving perinatal outcomes: the time to act. Maternal Child Health Journal..

[9] Bhutta Z, Dean S, Imam A, Lassi Z (2011). A systematic review of preconception risks and interventions..

[10] Chandra A, Martinez GM, Mosher WD, Abma JC, Jones J (2005). Fertility, family planning, and reproductive health of US women: Data from the 2002 National Survey of Family Growth. Vital Health Statistics..

[11] Johnson K, Posner SF, Biermann J (2006). CDC/ ATSDR preconception care work group: select panel on preconception care. Recommendations to improve preconception health and health care United States..

[12] Lynch J, Smith GD, Hillemeier M (2001). Income inequality, the psychosocial environment, and health: comparisons of wealthy nations. Lancet..

[13] Finch BK (2003). Early origins of the gradient: the relationship between socioeconomic status and infant mortality in the United States. Demography review..

[14] Cogswell ME, Yip R (1995). The influence of fetal and maternal factors on the distribution of birth weight. Journal of Semin Perinatology..

[15] Brown CA, Sohani SB, Khan K (2008). Antenatal care and perinatal outcomes in Kwale district, Kenya. BMC Pregnancy and Childbirth..

[16] Lawrence C E, Patrick Mbah, Jeanne Kouam (2007). Pre-conception care in Cameroon: Where are we now?. International Journal of Gynecology & Obstetrics..

[17] Abasiattai AM, Utuk NM, Udoma EJ (2011). Nigerian Journal of Medicine..

[18] Rezaeian M, Salem Z (2007). Prevalence of obesity and abdominal obesity: in a sample of urban adult population within South East of Iran. Pakistan Journal of Medical Science..

[19] Adedoyin RA, Mbada CE, Balogun MO (2009). Obesity prevalence in adult residents of Ile-Ife. Nigerian Quarterly journal of Hospital medicine..

